# Neuronal Autophagy: Regulations and Implications in Health and Disease

**DOI:** 10.3390/cells13010103

**Published:** 2024-01-04

**Authors:** Caroline Liénard, Alexandre Pintart, Pascale Bomont

**Affiliations:** 1 NeuroMyoGene Institute—PGNM, CNRS UMR 5261—INSERM U1315, University of Claude Bernard Lyon 1, 69008 Lyon, France; caroline.lienard@univ-lyon1.fr (C.L.); alexandre.pintart@univ-lyon1.fr (A.P.); 2 CHU Montpellier, University of Montpellier, 34295 Montpellier, France

**Keywords:** autophagy, regulations, compartmentalisation, neurodevelopment, neurodegeneration

## Abstract

Autophagy is a major degradative pathway that plays a key role in sustaining cell homeostasis, integrity, and physiological functions. Macroautophagy, which ensures the clearance of cytoplasmic components engulfed in a double-membrane autophagosome that fuses with lysosomes, is orchestrated by a complex cascade of events. Autophagy has a particularly strong impact on the nervous system, and mutations in core components cause numerous neurological diseases. We first review the regulation of autophagy, from autophagosome biogenesis to lysosomal degradation and associated neurodevelopmental/neurodegenerative disorders. We then describe how this process is specifically regulated in the axon and in the somatodendritic compartment and how it is altered in diseases. In particular, we present the neuronal specificities of autophagy, with the spatial control of autophagosome biogenesis, the close relationship of maturation with axonal transport, and the regulation by synaptic activity. Finally, we discuss the physiological functions of autophagy in the nervous system, during development and in adulthood.

## 1. Introduction

Autophagy is a major and evolutionarily conserved process that maintains cellular homeostasis through the degradation and recycling of cellular content, misfolded or damaged proteins and intracellular pathogens. In mammalian cells, three different types of autophagy coexist: macroautophagy, microautophagy, and chaperone-mediated autophagy. Macroautophagy (hereafter referred to as autophagy) is characterised by the engulfment of targeted cargos in a double-membraned vesicle, known as a phagophore, which expands to form a closed vesicle, the autophagosome. Autophagosomes then mature and fuse with lysosomes to form autolysosomes where their content is degraded [1].

As regulators of protein and organelle integrity, autophagy sustains various physiological functions that are essential for cell differentiation, development, survival, and aging; therefore, alterations in the autophagy pathway are responsible for a wide range of pathologies, including cardiovascular and pulmonary diseases, metabolic disorders, infections and inflammation, cancers, and neurodegenerative diseases [2,3,4]. Present in various tissues, autophagy undergoes differential regulation to sustain tissue-specific functions. One striking example is its strong response to starvation, a potent inducer of autophagy in muscle with a mild effect in brain tissues [5].

Unlike other cell types, neurons are post-mitotic cells that are incapable of discarding toxic components during cell division. Furthermore, neurons are highly polarised, with dendrites and an axon that can extend up to a metre in humans. With a specific morphology, neurons control specific functions, such as neurotransmission, neural circuits, and synaptic plasticity. Thus, neurons need robust and spatially orchestrated quality control mechanisms to ensure their homeostasis, and alterations in the autophagy pathway underlie numerous neurodevelopmental and neurodegenerative disorders [6,7,8].

In this work, we first review the highly orchestrated autophagy machinery and the increasing number of regulators that modulate each step of the process. We then focus on neuronal autophagy, to scrutinise its spatial compartmentalisation and its roles in neuronal development and physiology. Throughout the review, we will introduce neuronal pathologies that are either directly caused by mutations in core autophagic genes or by regulators of the pathway, and we will present how studies of the underlying pathophysiological mechanisms enlighten our understanding of this complex and highly ordered degradative pathway.

## 2. The Autophagy Pathway

**Figure 1:**
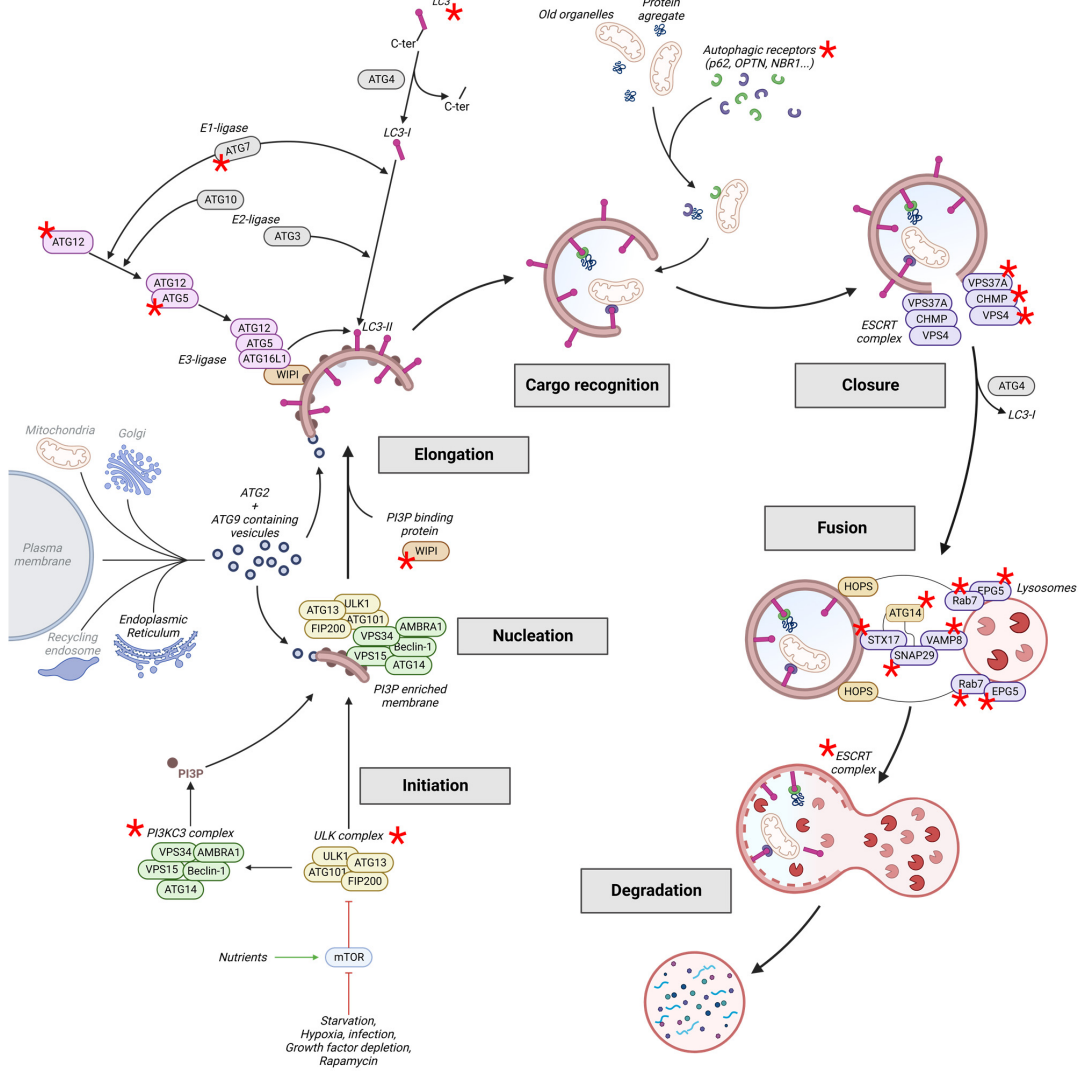
Schematic representation of the autophagic pathway. This process produces a double-membrane vesicle called an autophagosome. During its elongation, the autophagosome recognises and engulfs old cytoplasmic (organelles, proteins, etc.) and damaged material (organelles, proteins aggregates, etc.). After maturation, the intravesicular content is degraded due to the fusion with lysosomes and is re-used by the cells to produce new proteins and organelles. Autophagy is regulated by the autophagy-related (ATG) proteins and many other regulators. Core autophagy proteins mutated in neuronal pathologies are tagged by a red asterisk and are described in more detail in Table 1. OPTN: Optineurin; NBR1: Next to BRCA1 gene 1 protein; ESCRT: endosomal sorting complexes required for transport; CHMP: charged multivesicular body protein; HOPS: homotypic fusion and protein sorting complex; EPG5: ectopic P granules protein 5 homolog; VAMP8: vesicle-associated membrane protein 8; STX17: syntaxin 17; and SNAP29: synaptosomal-associated protein 29.

The first description of autophagy was as a bulk and non-selective degradative pathway induced by nutrient deprivation and glucagon [9,10,11,12]. Since then, autophagy has been shown to be modulated by other types of stress leading to the selective autophagy of specific cargos, including protein aggregates (aggrephagy), damaged organelles (mitophagy, pexophagy, ER-phagy, etc.) or pathogens (xenophagy) [13]. Autophagy is orchestrated by a complex cascade of events regulated by more than fifteen autophagy-related (ATG) proteins and various inducers and regulators (Figure 1). Mutations of these proteins are involved in a wide range of neurodevelopmental and neurodegenerative diseases (Table 1).

### 2.1. Induction: Initiation and Nucleation

The initial stage of autophagy is under the control of mTOR (mammalian Target Of Rapamycin) and is regulated by the Unc-51-like kinase (ULK) complex (comprising ULK1, focal adhesion kinase family interacting protein of 200 kDa (FIP200), ATG13, and ATG101), which plays a major role through the induction of a phosphorylation cascade of downstream effectors. Under nutrient-rich conditions, mTOR phosphorylates ULK1 and ATG13, hence suppressing ULK1 catalytic activity and leading to autophagy inhibition [14,15]. Moreover, phosphorylated ATG13 also negatively regulates ULK1 activity contributing to the inhibition of autophagy initiation. Thus, inactivation of mTOR that is induced by nutrient starvation or rapamycin treatment results in the dephosphorylation of ULK1 and ATG13, which induces ULK1 activity. Active ULK1 induces its auto-phosphorylation, which in turn activates the other components of the ULK complex, hence triggering this catabolic process [16]. After the process has been triggered, the ULK complex interacts through ATG101 with the nucleation complex PI3KC3 (class III phosphatidylinositol 3-kinase) composed of Beclin1 (BECN1), vacuolar protein sorting (VPS) 34 (VPS34), VPS15, ATG14, and the activating molecule in BECN1-regulated autophagy protein 1 (AMBRA1) [17]. ULK-mediated phosphorylation of PI3KC3 components promotes their stabilization and activity, to produce phosphatidylinositol-3-phosphate (PI3P) [18]. As detailed in the next section, other ATG proteins (like WD repeat domain phosphoinositide-interacting proteins (WIPIs), ATG16, and LC3 (microtubule-associated protein 1 light chain 3, the mammalian ortholog of Atg8)) are recruited to PI3P-enriched membranes at the phagophore assembly site during autophagosome biogenesis [19,20,21].

The nucleation of the phagophore is dependent on ATG proteins and membrane supply, which is mediated by ATG2 and ATG9. ATG2, assisted by WIPI proteins, mediates the transport of lipids to the phagophore, extracting them from vesicles to PI3P-containing membranes [22,23,24]. Lipid transfer is also promoted by ATG9, the only transmembrane ATG protein located in the phagophore, the trans-Golgi network and endosomes [25]. This ATG protein seems to shuttle between these organelles, providing membrane sources to the nascent autophagosome.

**Table 1:** Mutations in genes encoding for core autophagic proteins in neurodevelopmental and neurodegenerative diseases.

Autophagic Step	Protein Mutated	Pathology	Refs.
Induction and nucleation	FIP200	Psychiatric disorders	[26,27,28,29]
	AMBRA1	NDD with encephalocele, spina bifida and NTD	[30]
	VPS15	Cortical atrophy, Epilepsy	[31]
	VPS35	PD	[32,33,34]
Elongation	ATG7	NDD with cerebellar hypoplasia and ataxia, PD	[35,36]
	ATG5	PD, SCA25, Cerebral Palsy	[37,38,39]
	ATG12	PD	[40]
	WIPI2 WIPI4	NDD with mental retardation, BPAN	[41,42,43]
	LC3	PD (risk factor), ASD	[44,45]
Cargo recognition	ALFY	Microcephaly with ID	[46]
	p62/SQSTM1	NADGP, ALS, FTD, AD (risk factor)	[47,48,49,50,51,52,53,54]
	NBR1	PD, DLB, AD	[55]
	Optineurin	ALS	[56,57,58]
	Ubiquilin-2	ALS	[59,60]
Maturation: Closure and Fusion	VPS37A	HSP	[61]
	VPS4A	DD and ID	[62]
	CHMP1A CHMP2B	pontocerebellar hypoplasia with microcephaly, ALS, FTD	[63,64,65,66,67,68]
	Rab7	CMT type 2B	[69,70]
	EPG5	Vici syndrome	[71]
	WIPI3/4	NDD, BPAN	[41,42,43,72]
	SNX14	SCAR17/20	[73,74,75]

NDD: Neurodevelopmental disorder; NTD: neural tube defects; PD: Parkinson’s disease; SCA(R): spinocerebellar ataxia (recessive); BPAN: Beta-propeller Protein-Associated Neurodegeneration; ASD: Autism Spectrum Disorder; NADGP: Neurodegeneration with ataxia, dystonia, and gaze palsy, childhood-onset; ALS: Amyotrophic Lateral Sclerosis; FTD: Fronto-temporal Dementia; AD: Alzheimer’s disease; DLB: Dementia with Lewy Bodies; HSP: Hereditary spastic paraplegia; DD: Developmental delay; ID: Intellectual disability; and CMT: Charcot–Marie–Tooth disease.

Various proteins of the ULK1 initiation complex have been found to be mutated or dysregulated in neurological diseases, hence pointing towards an impairment of autophagy signalling in pathophysiology. Mutations or copy number variations in the gene encoding for FIP200 (*RB1CC1*) were identified in patients affected by psychiatric disorders with developmental delay (DD), autism spectrum disorder (ASD), and schizophrenia (SZ) [26,27,28,29]. The role of FIP200 deficiency in cerebellar and glutamatergic neurons has been confirmed in psychiatric disorders, with FIP200 depletion in neural progenitor cells and ULK1/2 inhibitor inducing axonal swellings, hyperactivity, and neuronal death [76,77]. FIP200 depletion was shown to cause impaired autophagic activity and abnormal accumulation of ubiquitinated protein aggregates in the central nervous system.

In the nucleation complex, the functional interaction between Beclin1 and VPS34 is promoted by AMBRA1, whose downregulation results in decreased autophagy *in vitro*, and total loss *in vivo* [78]. In humans, five rare missense mutations of AMBRA1 have been identified in 352 individuals affected by neurodevelopmental disorders (NDD) with neural tube defects, suggesting its role in neurodevelopment [30]. Through a loss-of-function mechanism, these mutations induce and alter the induction of autophagy *in vitro*. Autophagic disruption has also been described as the consequence of a hypomorphic mutation in VPS15, associated with cortical atrophy and epilepsy in humans. Moreover, ablation of VPS15 results in the accumulation of the autophagic receptor p62, reflecting a defect of autophagic degradation, thereby leading to the induction of apoptosis and neuronal loss in the cortex [31].

ATG9 trafficking to the phagophore can be altered in disease. In Parkinson’s disease (PD), mutations were identified in the *VPS35* gene [32,33], which is crucial for trafficking from the endosome to the trans-Golgi network and for the recycling of membrane proteins. A PD-causing dominant mutant of VPS35 is responsible for alterations in ATG9A trafficking, resulting in a smaller proportion of LC3-positive autophagic structures and contributing to decreased autophagy in cell lines expressing the PD-associated variant [34]. Interestingly, mutations in other genes controlling the ATG9A trafficking are also found in neurodegenerative diseases. Thus, the adaptor protein AP-4, mediating the ATG9A export from the trans-Golgi network to the phagophore assembly site, is mutated in Hereditary Spastic Paraplegia (HSP), an upper motor neuron disease [79]. AP-4 deficiency impairs ATG9A localisation and induces aggregate formation as a consequence of decreased autophagosome production in neurons, likely contributing to the development of the disease [80,81].

### 2.2. Elongation

#### 2.2.1. Membrane Expansion

Membrane expansion of the autophagosome is regulated by two ubiquitin-like reactions, which induce the lipidation of LC3 (Figure 1). Conjugation of ATG5 with ATG12 is mediated by ATG7 (E1-like) and ATG10 (E2-like) [82,83,84]. The ATG5-ATG12 complex then interacts with ATG16L1, which is recruited to the phagophore membrane by FIP200 [85] in stoichiometric proportions to form the ATG5-ATG12-ATG16L1 multimeric complex [86,87]. In parallel, LC3 is cleaved at its C-terminal portion by ATG4 to generate LC3-I [88]. Then, LC3-I is anchored to the nascent phagophore membrane by conjugating with a phosphatidylethanolamine, hence generating LC3-II. This reaction is mediated by ATG7 (E1-like), ATG3 (E2-like) and ATG5-ATG12-ATG16L1 (E3-like) [89,90,91]. The site of LC3 lipidation is determined by the ATG5-ATG12-ATG16L1 complex through the specific recruitment of ATG16L1 at the phagophore membrane by WIPI proteins (WIPI 1–4) [92].

Mutations in these core autophagy genes are causing several human neurodegenerative diseases (Table 1).

The *ATG7* gene is mutated in NDD presenting complex symptoms, including cerebellar hypoplasia, ataxia and abnormalities in the corpus callosum. Recessive mutations were shown to decrease the level of ATG7 protein and LC3 lipidation and to induce an accumulation of the autophagic receptor p62 [35]. Other studies have suggested that ATG7 has a role in PD. Specific *ATG7* deletion in cerebellar Purkinje cells or in dopaminergic neurons in mice induces progressive dystrophy manifested by axonal and dendritic swelling and degeneration of axon terminals with an accumulation of autophagosomal structures [36,93]. Neuronal death is accompanied by behavioural deficits such as locomotor dysfunction in mutant mice. Genetic and functional analyses of *ATG7* gene promoters in sporadic PD patients have highlighted variants inducing a significant decrease of the transcriptional activity [36], which is argued to impair autophagic activity.

ATG5 and ATG12 were also found mutated in sporadic PD patients [37,40]. Homozygous missense mutation in ATG5 is also responsible for spinocerebellar ataxia (SCA25). Cells from patients display a decrease in autophagy flux due to improper conjugation of ATG12 to ATG5 [38]. A different variant of *ATG5* has been identified in patients affected by Cerebral Palsy, a group of developmental disabilities. This mutation induces decreased protein level in the plasma [39], as seen in the *ATG5* null neurons which exhibit autophagy defects and neurodegeneration [94]. This mutation has been argued to downregulate ATG5 and interfere with autophagic activity [39].

Mutations in WIPI proteins are responsible for neurological disorders. WIPI4 mutations induce neurodegeneration with brain iron accumulation (NBIA). Lymphoblastoid cell lines derived from patients affected by β-propeller protein-associated neurodegeneration (BPAN, a subtype of NBIA) exhibit a decrease in autophagic activity with an accumulation of early autophagic structures due to WIPI4 protein instability [41]. This has been evidenced by a specific depletion of WIPI4 in the central nervous system of mice, which exhibit a defect in autophagic flux alongside p62 and ubiquitin-positive protein aggregation in neurons and swollen axons. These mice recapitulate some hallmarks of BPAN confirming the role of WIPI mutations in the disease [95]. Homozygous mutation in WIPI2 was identified in a patient with a complex developmental disorder presenting a wide range of symptoms, including mental retardation, speech and language impairment, and other neurological, psychiatric, skeletal, and cardiac abnormalities. Binding of the mutant WIPI2 to ATG16L1 (as well as ATG5-ATG12) was found to be significantly reduced. Fibroblasts derived from patients show less WIPI2 puncta, a decrease of LC3 lipidation, and reduced autophagic flux [42]. Another study presented a WIPI2 variant that leads to increased LC3 lipidation but with no difference in degradation efficiency [43].

Mutations within the promoter of the *LC3B* gene were identified as a risk factor for PD, which enhances its promoter activity, leading to increased levels of LC3B transcripts [44]. In addition, an elevation of the level of LC3B could be detected in leukocytes from PD patients, which exhibit enhanced LC3 lipidation [96]. LC3 and GABARAP (GABA receptor-associated protein, another mammalian ortholog of Atg8) mutations have also been found in ASD, but no data on autophagy are available [45].

Autophagosome formation is coupled with cargo detection, with targets engulfed in the vesicle before the closure of the phagophore. The adaptor protein Ubiquilin-2 (UBQLN2), mutated in ALS and FTD [59,60] has been shown to interact both with LC3 [97,98] to control conversion of LC3-I to LC3-II [98] and with protein aggregates [99,100], particularly K48- and K63-ubiquitinated proteins in autophagosomes [98] (see details below).

#### 2.2.2. Cargo Recognition

In selective autophagy, cargos are targeted and directed to the autophagosome by autophagic receptors: p62/SQSTM1, Optineurin (OPTN), NBR1 (Next to BRCA1 gene 1 protein), NDP52 (also known as Calcium-binding and coiled-coil domain-containing protein 2, Calcoco2), and Ubiquilins [101,102]. This action by the autophagic receptors is mediated by their Ubiquitin Binding Domain (UBD) (or Ubiquitin Associated Domain: UBA) that recognises ubiquitinated cargo and by their LC3-interacting region, which permits the addressing to the autophagosome.

Among the autophagic receptors, p62 is the most commonly described [103,104,105]. In selective autophagy, autophagosomes can be formed around a targeted cargo. In this situation, p62, bound to aggregate, can directly interact with ALFY (autophagy-linked FYVE protein), a PI3P binding protein [106,107,108]. As ALFY also binds to ATG5-ATG12, this adaptor protein connects the different components permitting ATG5-ATG12-ATG16 to expand the membrane around p62-containing aggregates [108]. Thus, cargo recruitment and phagophore formation are dependent on each other. This may explain why depletion of p62 inhibits the recruitment of LC3 to autophagosomes under starvation conditions [103].

Mutations in autophagic receptors have been evidenced in several neuropathies [109]. In humans, dominant mutations in ALFY cause primary microcephaly [46]. Mutations in the *Sqstm1* gene (encoding for p62) are found in ALS and FTD [48,49,50,51]. Also, loss-of-function mutations in *Sqstm1* have been identified in patients affected by neurodegeneration with ataxia, dystonia, and gaze palsy, childhood-onset (NADGP), characterised by gait abnormalities, dysarthria, and cognitive decline. Mutant fibroblasts of these patients exhibit a decrease in autophagosome production [47].

A mega meta-analysis of existing genome-wide association studies on Alzheimer’s disease (AD) identified p62 variants that may represent a risk factor for the pathology [54]. While the causal role of p62 remains to be confirmed, p62 and NBR1 accumulate in the striatum and hippocampus of HD mice and patients [110]. NBR1 was also found accumulated in brains from patients affected by PD, dementia with Lewy bodies, and AD [55].

Other autophagic receptors are also associated with the same neurodegenerative diseases. Mutations within the *OPTN* gene were identified in sporadic ALS and non-SOD1 familial ALS [56]. Ubiquilin-2, which can be considered as an autophagic receptor since it binds LC3 on autophagosome and ubiquitinated proteins [97,98,99], has been found mutated in ALS/FTD patients [59,60]. ALS/FTD UBQLN2 mutants were shown to disrupt autophagy, with accumulation of p62 and ubiquitinated proteins and decreased autophagic degradation [111]. Interestingly, mice carrying UBQLN2 mutations develop age-dependent motor neuron disease and cognitive deficits [112].

### 2.3. Closure and Fusion

#### 2.3.1. Closure

The autophagosome is formed after membrane extension and fusion. Several ATG proteins were reported to be involved in autophagosome closure [113]. ATG3, an essential factor in membrane elongation, is also required for the dissociation of ATG5-AT12-ATG16L1 from the membrane to promote fusion. Indeed, ATG3 depletion reduces not only the elongation of phagophore membranes but inhibits the full closure of the autophagosome [114]. In the same way, ATG5-deficient cells can produce phagophore but not closed vesicles [20]. Silencing of *ATG2* induces a similar phenotype with a block in autophagic flux and an accumulation of unclosed autophagic structures [115]. This autophagosome closure defect is most likely due to a decreased interaction between ATG2 and proteins from the GABARAP family, such as LC3, which is essential for phagophore maturation. Indeed, it has been demonstrated that a loss of this interaction results in the accumulation of open and immature vesicles and a complete blockage of autophagy flux [116].

Autophagosome closure requires several endosomal sorting complexes required for transport (ESCRTs). ESCRT-I is recruited to the phagophore membrane through its subunit VPS37A [117]. VPS37A promotes the assembly of the charged multivesicular body protein (CHMP) 2A (CHMP2A), a component of the ESCRT-III complex, which then recruits the AAA-ATPase VPS4 [117]. CHMP2A and VPS4 are necessary for the fission of outer and inner membranes of the autophagic vesicle (AV) and autophagosome closure. Thus, inhibition or depletion of VPS37A, CHMP2A, and VPS4 induce an autophagosome closure defect [117,118]. Accumulations of unsealed AVs harbouring ATG proteins are observed in cells depleted of the ESCRT-III subunit snf7 or VSP4 [119]. Upon vesicle closure, LC3 and GABARAP proteins are removed from the outer membrane of the autophagosome by ATG4-mediated cleavage [88].

Factors implicated in autophagosome closure are responsible for neurological disorders. Mutation in VPS37A has been identified in HSP [61]. Missense mutations in VPS4A are implicated in a NDD characterised by severe hypotonia, DD, and intellectual disability (ID) [62]. CHMP1A and CHMP2B, two subunits of the ESCRT-III complex involved in autophagy [66], are mutated respectively in autosomal-recessive pontocerebellar hypoplasia with microcephaly and ALS/FTD [63,65]. Depletion of these ESCRT subunits leads to the accumulation of unclosed AVs where ULK1, ATG5 and p62 accumulate, resulting in an impaired autophagic flux [117,118].

#### 2.3.2. Fusion

After their completion, closed autophagosomes mature into autolysosomes by fusing with lysosomes, which permits the degradation of autophagocytosed cargos. This final step of autophagosome maturation begins with the movement of both vesicles to enable their contact. This is mediated by ATG proteins and the SNARE (synaptosomal-associated protein receptor) and HOPS (homotypic fusion and protein sorting) complexes. The SNARE complex is composed of two parts, one being present on the autophagosome (t-SNARE: Syntaxin 17 (STX17) and SNAP29) and the other on the lysosome (v-SNARE: vesicle-associated membrane protein (VAMP) 8 (VAMP8)) [120,121]. The formation of SNARE complex is regulated by ATG14, which promotes membrane tethering via the t-SNARE and v-SNARE [120]. The action of ATG14 depends on its homo-oligomerisation [122] and is mediated by its binding to the STX17-SNAP29 binary complex on autophagosomes [123]. However, the SNARE complex is not able to induce autophagosome-lysosome fusion alone and requires another complex. The contact between an autophagosome and a lysosome is mediated by the HOPS complex, Rab7 and its effector ectopic P granules protein 5 (EPG5). EPG5 promotes fusion by binding to Rab7, VAMP7/8, and assembled t-SNARE complexes [124], and its deficiency in *Caenorhabditis elegans*, mice, and humans has been shown to inhibit autolysosome formation [71,125,126]. Thus, the HOPS complex bridges the gap between the tightly apposed autophagosomal and lysosomal membranes and facilitates the assembly of the trans-SNARE complex to mediate fusion [124]. Finally, membrane fusion between the autophagosome and lysosome is dependent on ESCRT complexes [127]. Depletion or inhibition of ESCRT subunits induce autophagosome accumulation [66,67].

Missense mutations in the small GTP-ase Rab7 cause CMT2B [69]. Dermal fibroblasts derived from CMT2B patients present reduced autophagic flux and lysosomal degradation, characterised by autophagosome and lysosome accumulations due to fusion inhibition [70,128]. Mutations in the *EPG5* gene are causal for Vici syndrome, a rare autosomal disease characterised by agenesis of the corpus callosum with severe psychomotor retardation and seizures [71,129]. Moreover, depletion of WIPI3/4, mutated in NDD and BPAN, has been shown to induce EPG5 mislocalisation, which impairs autophagosome fusion with late endosomes/lysosomes [72].

The possible role of the sorting nexin 14 (SNX14) in fusion has emerged from studies on disease models. SNX14 mutations lead to SCA17/20 and SCA-like phenotypes [73,74,75]. Also, snx14 zebrafish and SCAR17 patient-derived cells induce a slower autophagic degradation upon starvation, characterized by engorged lysosomes and the presence of p62 positive vacuoles [73], suggesting an impairment of autophagosome-lysosome fusion.

The ESCRT-III subunits CHMP -1A and -2B, also essential for autophagosome-lysosome fusion, are found mutated in neurodegenerative diseases such as ALS and FTD [66,67,68].

## 3. Compartmentalisation of Neuronal Autophagy

**Figure 2:**
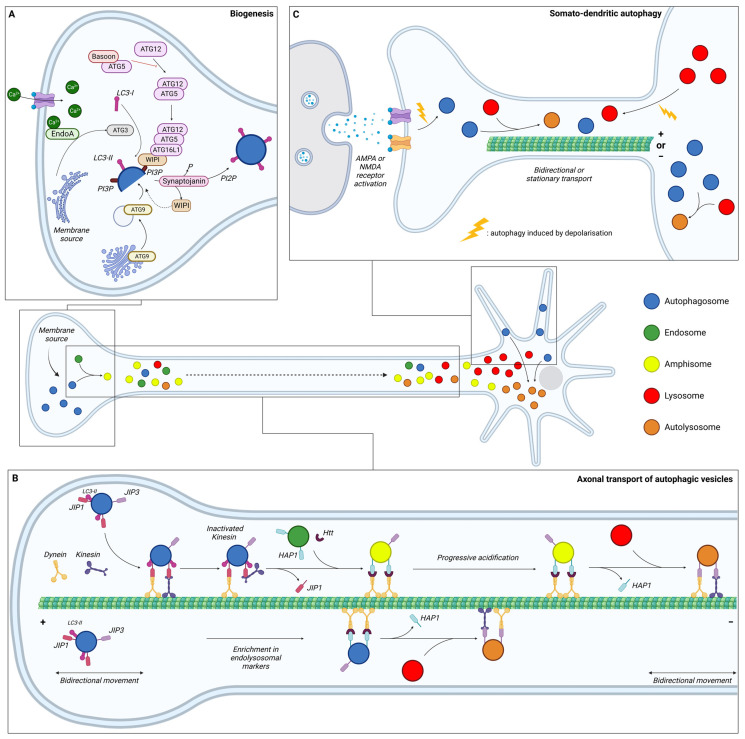
Schematic representation of the dynamics of neuronal autophagy. (**A**) Autophagosome (blue) biogenesis takes place in the distal part of the axon. (**B**) After their formation, autophagosomes first undergo bidirectional movement and switch to retrograde transport to move towards the soma along microtubules with the help of motor proteins (dynein and kinesin). During this transport, autophagic vesicles (AVs) mature by fusing with endosome (green) and/or lysosome (red) to generate, respectively, amphisome (yellow) and/or autolysosome (orange). In the proximity of the soma, AVs become more mature and exhibit bidirectional movement. (**C**) Dendritic autophagosome biogenesis is regulated by synaptic activity. Depolarisation, induced by KCl or through AMPAR/NMDAR, increases the density of AVs in dendrites and decreases displacement. Lysosomes from the soma are also recruited to dendrites after depolarisation. In somal autophagy, AVs tend to be less mobile than in axons or dendrites. This compartment is the main site of final autolysosome content degradation. Ca: calcium; EndoA: endophilin A; JIP: JNK-interacting protein; Htt: Huntingtin; HAP1: Huntingtin-associated protein 1; AMPA: α-amino-3-hydroxy-5-methyl-4-isoxazolepropionic acid; and NMDA: N-methyl-D-aspartate.

Neurons are specialised and polarised cells with three different compartments: the soma, the dendrites, and the axon. To sustain specialised functions, neuronal protein composition must be tightly regulated in each compartment, from synthesis to degradation. Moreover, given their post-mitotic nature, neurons cannot dilute toxic components through cell division and are therefore highly dependent on efficient degradative pathways. It is considered that in contrary to other cell types, neurons continuously generate autophagosomes under basal conditions. Neurons undergo compartment-specific autophagy regulation [130] (Figure 2). Studies have revealed that autophagosome biogenesis occurs in the distal part of the axon; maturation occurs during retrograde transport, while their content is degraded in the soma upon fusion with lysosomes. Interestingly, another site of biogenesis has been identified in the somatodendritic compartment, but their dynamics and functions are poorly understood.

### 3.1. Autophagy in the Axon

#### 3.1.1. Biogenesis

Initial research suggested that autophagosome formation occurs in the distal part of the axons (Figure 2A). Indeed, dense-cored vesicles, formed at least in part from the reticulum, were described in the growth cone of isolated sympathetic neurons *in vitro* [131]. Identified as AVs containing cytoplasmic material, organelles were shown to be mobile through retrograde transport in the axon of peripheral neurons [132]. Using the GFP-LC3 probe in live imaging, ring-like structures of approximately 800 nm in diameter were found accumulated near the distal end of the axon [133]. These vesicles were identified as autophagosomes based on their strong LC3-positive signals and the similarity in morphology with the LC3-positive structures seen *in vivo* in transgenic GFP-LC3 mice [5], indicating de novo formation of autophagosomes in the distal tip.

In contrary to non-neuronal cell types, whose membranes can be of multiple origin, endoplasmic reticulum (ER) is the major source of membrane for nascent autophagosomes in neurons. More specifically, AVs emerge from specialized subdomains of the ER labelled by double-FYVE-containing protein 1 (DFCP1). As discussed in the first part of this review, autophagosome formation is dependent on the transmembrane core protein ATG9. This lipid scramblase derives from the trans-Golgi network and mediates the translocation of phospholipids between two lipidic membranes, thus providing a phospholipid source for autophagosomal membrane expansion [134]. Thus, mislocalisation of ATG9A or its retention in the trans-Golgi network, as seen in HSP, reduces autophagosome formation [80,135]. Regulating exocytosis and endocytosis, ATG9 is also implicated in synaptic activity and its mislocalisation is associated with defects in activity-induced synaptic autophagy [136,137]. In *C. elegans*, the synaptic protein Clarinet (CLA-1L), bearing similarity to the vertebrate Rab-interacting molecule (RIM), Piccolo, and Bassoon, regulates the localisation of ATG-9 at presynaptic sites, thus promoting autophagy during neuronal activity. Indeed, *Cla-1(L)* mutants were found to disrupt ATG-9 sorting and to induce deficits in autophagosome formation at the synapses [138].

Autophagosome biogenesis in the distal tip of the axon involves the same ordered recruitment of the core autophagic proteins as in non-polarised cells [139,140,141]. Nevertheless, some cell-type specificity occurs in neurons, with specialised proteins of the presynapse playing a role in the regulation of AV biogenesis. First, Bassoon binds to ATG5 and negatively regulates the ATG5-ATG12-ATG16 E3 ligase activity to suppress synaptic autophagy [140]. Thus, loss of Bassoon and Piccolo is associated with a local induction of autophagy, which contributes to the destruction of synaptic vesicles through the fusion of bouton-derived autophagosomes with somatic lysosomes. Secondly, the recruitment of ATG proteins at the synapse is calcium-dependent and mediated by phosphorylated Endophilin A (EndoA). Curved membranes induced by phosphorylated EndoA allows for the recruitment of ATG3, promoting LC3 lipidation and thus autophagosome formation [142,143]. To allow the local and constant biogenesis of autophagosomes, synaptojanin, a lipid phosphatase enriched in synaptic boutons, dephosphorylates PI3P to enable WIPI2 removal from nascent phagophores [144]. This recycling replenishes the WIPI2 soluble pool to form new phagophores.

In PD, EndoA phosphorylation has been described as deregulated due to mutations in the leucine-rich repeat serine/threonine-protein kinase 2 (LRRK2) [145]. LRRK2 mutation inhibits EndoA membrane association, endocytosis, and autophagy, leading to dopaminergic neuron degeneration [142,146]. The role of LRRK2 has been confirmed in flies expressing a phospho-deficient EndoA, which exhibit an inhibition of starvation-induced synaptic autophagy [142]. Moreover, EndoA mutation has also been identified as a PD risk variant and was shown to be mislocalised and to block the formation of autophagosomes at pre-synaptic terminals after stimulation and Ca^2+^ influx [147]. Another PD risk mutation in EndoA at the SH3 domain containing GRB2 like 2 (SH3GL2) has been described as disrupting the calcium sensing of SH3GL2. The resultant immobile protein cannot respond to calcium influx and therefore disrupts autophagy induction at the synapses [148]. A PD mutation in the SCA1 domain of synaptojanin inhibits its phosphatase activity. Mutant flies and iPSC-derived neurons from patients exhibit accumulation of WIPI proteins (Atg18 in flies) on nascent synaptic autophagosomes. This inhibits their completion, leading to their accumulation in the synapses [144].

#### 3.1.2. Maturation during Transport

After their formation at the distal part of the axon, autophagosomes mature by fusing with acidic vesicles, such as early and late endosomes, to form amphisomes and with lysosomes to form autolysosomes (Figure 2B). The situation is complex, with different fusion events generating distinct AVs all along the axons. To summarise, autophagosomes formed in the growth cone first undergo bidirectional movement [149], which then switches to unidirectional movement along the axon. This change in motility depends on the maturation state of the autophagosome. The acquisition of endo-lysosomal markers such as Rab5, Rab7 and LAMP1, indicating a fusion with endosome or lysosome, is necessary for dynein-mediated retrograde transport in axons [149,150,151]. P62 has also been reported to be required for proper dynein motility and trafficking along microtubules (MTs) via a direct interaction with dynein [152]. Conversely, knock-down of p62, as well as pharmacological inhibition of dynein activity with ciliobrevin D, were found to reduce autolysosome formation [153]. Thus, the maturation process is coupled with axonal transport and generates a spatial gradient of organelle pH along the axon [154]. Most organelles in distal axons (86%) are neutral with a pH of >6.8, while the first 100 µm of the axon, corresponding to the proximal part, comprises 44% of neutral organelles and 56% acidic with a pH of <6.0. The dual-colour autophagic probe mCherry-GFP-LC3, which labels immature AVs in yellow and mature AVs in red due to GFP quenching in an acidic environment, has allowed for research into maturation and transport coupling [149]. This analysis revealed that immature AVs are more abundant in the distal part of the axon compared to the proximal part, which is mostly composed of mature AVs.

In the neuron, axonal transport of AVs, coupled with maturation, depends on MTs, the molecular motors dynein and kinesin, and adaptors. In a nutrient-deprived environment, the addition of depolymerising drugs such as nocodazole prevents degradative response by inhibiting the delivery of lysosomal hydrolytic enzymes to autophagosomes [155]. Furthermore, live imaging of autophagosome dynamics with GFP-LC3 confirms that MTs are required for their motility and fusion with lysosomes [156].

Autophagosomes, formed in the growth cone, initially undergo a bidirectional movement along MTs with the help of kinesin and dynein [149]. Little is known about autophagosome motility regulation in the distal tips prior to their exit. Disruption of the retrograde transport by impairing the recruitment of dynein adaptors can induce kinesin activation and bidirectional or anterograde transport [157,158,159]. Moreover, the LC3B phosphorylation state regulates the directional transport of AVs. Its phosphorylation by the serine/threonine-protein kinase 4 (STK4) promotes the recruitment of the FYVE and coiled-coil domain-containing protein 1 (FYCO1), a kinesin adaptor for anterograde transport [160,161].

The switch of mobility in the distal axon, from bidirectional/stationary to unidirectional retrograde transport is poorly understood. Studies suggest that fusion between autophagosomes and endosomes in the distal tip plays a role in dynein recruitment [151,162,163]. To allow for their exit from the distal tips through unidirectional retrograde transport, dynein is recruited to AVs through interaction with JNK-interacting protein (JIP) 1 (JIP1), a motor adaptor [157,164]. While JIP1 can bind either dynein or kinesin, its interaction with dynein and LC3 induces an inactivation of kinesin, therefore promoting retrograde transport from the distal to the middle part of the axon [157,165].

AV retrograde transport in the middle axon has been shown to depend on the Huntingtin-associated protein 1 (HAP1) and HTT [158]. HAP1 forms a motor complex with dynein and dynactin via conserved dynein- and dynactin-binding sites [159]. This dynein–dynactin–HAP1 complex interacts with AVs via the scaffolding protein HTT. Depleting HTT in neurons of dorsal root ganglion results in a significantly decreased percentage of retrograde AVs and a significant increase of bidirectional or stationary movements [158]. Loss of HAP1 also decreases the run speeds of retrograde-directed AVs. Likewise, abolishing HTT–HAP1 interaction disrupts autophagosome transport, resulting in more stationary AVs [158].

Finally, as the AVs move proximally, another motor protein adaptor, JIP3, initially known to be involved in endosome transport, regulates the retrograde transport of acidic AVs via dynein interaction [159,166,167,168,169]. JIP3 mutants generate an increased number of AVs in neurites in *C. elegans*, zebrafish, and cortical neurons due to defective retrograde transport [166,167,168,169]. These motor adaptors are recruited in an AV maturation-dependent manner. JIP1 and HAP1 have been found to essentially bind with immature AVs, while JIP3 associates with both mature and immature AVs [159], confirming the coupling of the transport and maturation of AVs in neurons. Mature AVs, mostly found in the proximal axon, were shown to exhibit more stationary/bidirectional or anterograde movements than immature AVs [149]. After entry into the soma, AVs seem to be unable to re-enter the axon, therefore promoting fusion with the degradative lysosomes that are enriched in the soma [130].

Alterations in the maturation–transport coupling can be deleterious and cause neurodegeneration in humans.

First, defects in the maturation of AVs were found to cause an impairment of retrograde transport. Indeed, the blocking of autophagosome-lysosome fusion by STX17 depletion was shown to reduce the recruitment of dynein motors to AVs, thus immobilising them in axons [151]. Reduction of STX17 protein levels in the NDD named Koolen–de Vries Syndrome (KdVS) leads to an accumulation of autophagosomes in the excitatory synapses [170,171]. This suggests that the acidification of autophagosomes may be required to achieve long-distance and exclusive retrograde movement. In AD brain samples, dystrophic neurites exhibit an abnormal accumulation of immature AV forms, including in the synaptic regions. This suggests a defect in the maturation and/or transport of AVs in affected neurons [172]. In an AD model, soluble Aβ associates with amphisomes in the distal axon, while Aβ1–42 interferes with the assembly and recruitment of the dynein complex [151] through a direct interaction with dynein [173]. In AD mice, decreased transport leads to the formation of amyloid plaque, which amplifies the axonal transport defect [168,173]. Alteration of the degradative capacity of AVs also has an influence on their transport. Thus, treatment with cathepsin or acidification inhibitors was shown to alter axonal transport of AVs and lead to AD-like axonal dystrophy [150].

Secondly, the disruption of axonal transport through motors, adaptors, and cytoskeleton can have pathogenic outcomes. Thus, dynein loss-of-function by downregulation, pharmacological agent, or mutations found in patients constitutes pathogenic mechanisms of neurodegenerative diseases such as ALS, PD, and HD [174,175,176]. In humans, mutations in dynein cause malformations of cortical development with microcephaly, spinal muscular atrophy, and CMT [177,178,179]. HTT–HAP1 interaction has been found to be inhibited by polyQ-HTT, which disrupts the axonal transport of autophagosomes in HD mice [158]. As we discussed with the impact of LRRK2 mutations in the biogenesis of autophagosomes in PD [145], kinase activity is also involved in autophagosome transport. LRRK2 mutants were shown to reduce the axonal transport of autophagosomes in a kinase-dependent manner [180]. Hyperactive LRRK2 was shown to recruit the kinesin-activating motor adaptor JIP4 to the autophagosomal membrane. JIP4 recruitment induces abnormal kinesin activation, leading to non-processive anterograde and retrograde movements [180]. Neurons expressing a more hyperactive LRRK2 mutant exhibit more severe transport deficits than those previously described, suggesting that increased disruptions in autophagosome transport correlate with higher levels of LRRK2 kinase activity [181]. Moreover, cytoskeletal defects in numerous neurological disorders can alter the axonal transport of AVs [182,183]. In CMT, the MT-based transport is damaged by the enhanced binding of mutant heat shock protein beta-1 (HSPB1) to tubulin [184]. The increased binding of mutant HSPB1 to α-tubulin has been shown to inhibit AVs and protein aggregate transport along MTs [185]. In addition, HSPB1 mutants have been found to interact with p62 [186], which, in addition to its role in AV formation, has been found to promote AV motility and trafficking along MTs via a direct interaction with dynein [152]. Thus, the altered transport of AVs in CMT could be due to both MTs and p62 dysregulations.

#### 3.1.3. Degradation

The primary site of autophagosomal degradation is the soma, where lysosomes are concentrated (see part B). However, studies have revealed that lysosomes can also be present in the axons and fuse with autophagosomes outside the soma.

Lysosomes, labelled by LAMP1, have been observed in the axon and accumulate within the swollen axons of amyloid plaques in AD mice [168]. Often deficient in the main proteases (cathepsins B, D and L), these organelles have been argued to represent an early stage of lysosome maturation. They would result from the fusion of lysosomal precursors in the distal regions of axons, and, like autophagosomes, their maturation would require retrograde transport to the cell body. Indeed, luminal proteases are enriched in lysosomes present in the soma [187]. On the other hand, time-lapse imaging performed in microfluidic devices provides evidence for the presence of cathepsins D, B, and L in lysosomes within the axonal compartment. The anterograde transport of mature lysosomes formed in the soma, as seen by the movement of somal degradative lysosomes towards distal axons [188], is most likely sustaining the local degradation capacity at distal regions of the axon and at the synapse. Thus, disrupting the axonal delivery of degradative lysosomes through depletion of Arl8, a lysosomal kinesin adaptor, has been shown to induce axonal autophagic stress with accumulation of autophagosomes in distal axons [188]. The anterograde transport of mature lysosomes into the axon is facilitated by ER tubules, which promote kinesin-1-powered lysosome fission (as seen in autophagic lysosome reformation). ER tubule–lysosome contact has been reported to occur at the pre-axonal region, facilitating the entry of lysosomes in the axon. Disruption of these interactions causes an accumulation of enlarged and less motile lysosomes in the soma, without affecting their degradative capacities [189]. Altogether, these findings indicate that degradative lysosomes are dynamically delivered to distal axons during the development and maturation of neurons.

### 3.2. Autophagy in the Soma

Soma is the main site for degradation of autophagosome content, as degradative lysosomes are concentrated in the perinuclear area. As mentioned above, non-degradative lysosomes can be observed in all regions of neurons but competent lysosomes, identified by the activity of lysosomal hydrolases such as cathepsin D, are enriched in the soma. More precisely, 45% of LAMP1-labeled organelles in the soma contain cathepsin D. This number drops to 29% in dendrites and 30% in axons, suggesting that the percentage of degradative LAMP1 organelles is higher in the soma than in neuronal processes [190].

Interestingly, a population of large LC3-positive autophagosomes, exhibiting weak LAMP1 staining, has evidenced a novel biogenesis site for autophagosome in the soma [130]. Those structures were found to be stationary or oscillating within a small area of a few micrometres. Starvation does not increase the number of this autophagosome population. However, degradation inhibition with bafilomycin increases GFP-LC3 puncta in the soma but not in the axon. Very little is known regarding the molecular mechanisms and physiological roles of this specialised site of autophagosome biogenesis, but its existence is supported by studies performed on gigaxonin. This E3 ubiquitin ligase, mutated in giant axonal neuropathy (GAN) [191], was shown to control autophagosome elongation through ubiquitin-dependant degradation of ALTG16L1 [192]. In GAN neurons, the net production of autophagosomes is reduced as a result of ATG16L1 accumulation, whose location in the soma suggests a biogenesis site within this neuronal compartment [192].

Independent studies have shown that damaged or depolarised mitochondria within the axon are transported retrogradely towards the soma, where they are engulfed in newly formed autophagosomes and degraded [193,194].

### 3.3. Autophagy in Dendrites

Autophagy in dendrites has not been described as much as axonal autophagy.

Autophagosome biogenesis in dendrites is infrequent, with a weak AV density [120,167], indicating that autophagosomes observed in the soma mainly originate from the axon [139].

Autophagic activity in dendrites does not respond to starvation [130], but to neuronal activity. KCl-induced depolarisation induces an increase of the LC3-II/LC3-I ratio in cultured hippocampal neurons in a NMDAR- (N-methyl-D-aspartate receptor-) dependent manner and an increase of GFP-LC3 puncta in the soma and dendritic spines [195]. Activation of autophagosome biogenesis in dendrites has also been reported upon long-term depression with NMDA [196].

In contrast to the preferred retrograde transport in axons, autophagosomes in dendrites predominantly exhibit bidirectional transport [139]. In contrary to the axonal compartment, MTs within dendrites are equally oriented in each direction, which may explain the bidirectionality of AV transport [197]. KCl-induced depolarisation of neurons or pharmacological stimulation results in a very striking and rapid decrease of AV motility within dendrites by reducing their average speed [198]. Conversely, silencing synaptic activity with antagonists of excitatory AMPAR (α-amino-3-hydroxy-5-methyl-4-isoxazolepropionic acid receptor) and NMDAR induces AV motility in dendrites. This effect of neuronal activity on AV motility is specific to dendrites. Live imaging reveals that stimulation of synaptic activity increases the percentage of AVs at the synapse, whereas its silencing decreases association of AVs to post-synaptic regions [198]. AV motility in dendrites dependents on dynein since treatment with Ciliobrevin-D, an inhibitor of dynein-mediated transport, increases the density of dendritic AV after synaptic stimulation [196].

Inhibition of autophagosome/lysosome fusion induces an increased autophagosome density in the dendritic shaft of pyramidal neurons, which is amplified after NMDA treatment. This observation confirms that autophagosomes are present in dendrites even under basal conditions and suggests that their clearance upon fusion with lysosomes limits their detection [195]. Indeed, dendritic autophagosomes are described as rapidly acquiring LAMP1, likely through fusion with late endosomes and lysosomes [198]. Furthermore, stimulation of synaptic activity increases the number of acidic organelles such as lysosome in dendrites [198,199]. This suggests that the increase of lysosomes after NMDA treatment may participate in the rapid dynamics of autophagic degradation [195]. As in the axon, transport and maturation of AVs in dendrites are interlinked, and inhibition of lysosome protease activity decreases their motility in dendrites [199].

The degradative abilities of acidic organelles have been confirmed by DQ-BSA (dye quenched-bovine serum albumin), an endocytosed cargo that fluoresces only upon proteolytic cleavage. While DQ-BSA–positive puncta are scarce in axons, they have been found to be present in dendrites and enriched in the soma [198]. This confirms the degradative ability of LAMP1-positive vesicles containing cathepsin-B observed in the soma and in the proximal portion of dendrites [200]. However, LAMP1-positive compartments further than 25 µm from the soma were rarely found to contain cathepsin-B. Those observations indicate that in the distal dendrites the degradative capacity is not sufficient for terminal degradation, which, similarly to the axon, occurs mostly in the soma [200].

## 4. Autophagy in Neurological Physiopathology

**Figure 3:**
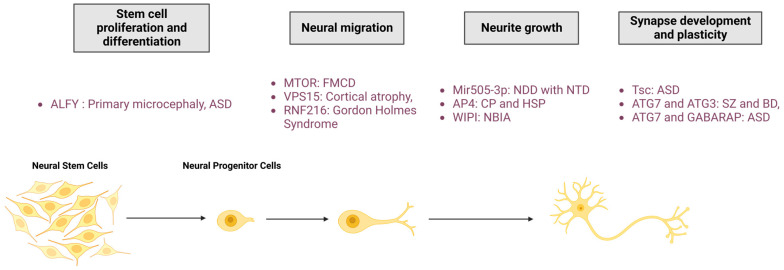
Roles of autophagy in neuronal physio(patho)logy. The autophagic process is fundamental for neuronal homeostasis and is implicated in stem cell proliferation and differentiation, neural migration, neurite outgrowth, and synapse development and plasticity. Mutation of proteins involved in autophagy pathway regulation leads to neurodevelopmental diseases. FMCD: Focal malformations of cortical development; NDD: Neurodevelopmental disorder; CP: Cerebral palsy; HSP: Hereditary spastic paraplegia; NBIA: Neurodegeneration with brain iron accumulation; ASD: Autism spectrum disorder; SZ: Schizophrenia; BD: Bipolar disorders; RNF216: Ring Finger Protein 216; Mir505-3p: miRNA 505-3p; and Tsc: tuberous sclerosis protein.

As demonstrated in the first two parts of this review, autophagy is an essential process for maintaining neuronal integrity. The initial evidence for the pivotal role of autophagy in nervous systems was found in mice, where neuronal depletion of ATG5 and ATG7 causes neurodegeneration with behavioural deficits, axonal swelling, and dendritic atrophy [93,94]. Moreover, deregulations in the autophagy pathway cause numerous NDDs, suggesting the role of this degradative pathway in neuronal development. In this third section, we review our current knowledge of the main roles of autophagy in neurological physiology, from stem cells homeostasis and neuronal development to synapse function and plasticity (Figure 3).

### 4.1. Neural Proliferation and Differentiation

Neurodevelopment in mammals is a complex process which starts in the neural stem cells (NSCs). They undergo symmetric cell division for self-renewal, proliferation to maintain stem cells niches, and asymmetric cell division to generate neural progenitor cells (NPCs). These progenitors are also able to self-renew and differentiate to produce neurons. Neurogenesis occurs during embryonic development and, to a lesser extent, in adulthood [201,202]. This process is regulated by numerous pathways, including autophagy [203].

Autophagy is necessary for maintaining NSC homeostasis and to control cell division. Indeed, autophagy has been shown to control the maintenance of NSCs in a quiescence state, since autophagy inhibition through ATG7 and ATG16L1 downregulation impairs differentiation and decreases neurogenesis [204]. These effects can be reversed through Beclin1 overexpression or under starvation. Epithelial v-like antigen 1 (Eva1a), a negative regulator of mTOR, is an important protein for NSC homeostasis, as Eva1a-depleted mice exhibit an inhibition of autophagy and an impairment of NSC self-renewal and differentiation in the cortex [205].

A premature transition between the symmetrical and asymmetrical division of NSCs leads to a decreased number of cortical neurons in the developing brain and causes microcephaly, a congenital NDD characterised by a reduced brain volume. This transition of cellular division is dependent on the autophagy adaptor protein ALFY [46]. Two different mouse models depleted for ALFY were shown to exhibit increased neural progenitor proliferation with increased cerebral size and a thinner neocortex in embryos [206,207]. Surprisingly, in humans, proliferation defects result in both microcephaly due to ALFY mutation [46] or macrocephaly due to *WDFY3*- (encoding for ALFY) haploinsufficiency, which has been associated with ASD [208,209] (Figure 3). Drosophila that express ALFY mutations found in patients affected by microcephaly have been described with reduced autophagy and premature asymmetric division leading to a reduced NCS pool [46].

The autophagy pathway plays a crucial role in NSC differentiation into NPCs and neurons. Protein levels of Beclin1, AMBRA1, ATG7 and LC3 have been found to progressively accumulate in the mouse embryo olfactory bulb during neuronal differentiation [210]. Moreover, inhibition of autophagy in mice through *BECN1*- (encoding for Beclin1) haploinsufficiency or AMBRA1 deficiency inhibits the differentiation of NSCs into NPCs, both in embryos and adults [210,211]. In adults, FIP200 has been found to be required for the maintenance and differentiation of postnatal NSCs. FIP200-depleted models show subventricular zone and dentate gyrus defects associated with impaired neurogenesis [212,213]. Autophagy inhibition by ATG5 downregulation increases the proliferation of cortical NPCs and inhibits their differentiation into neurons [214]. Additionally, the loss of ATG5 inhibits polarity acquisition of neural progenitors, which exhibit fewer and shorter neurites in the developing cortex.

### 4.2. Neural Migration

In non-neuronal and neuronal cells, motility requires assembly and disassembly of the focal adhesion. Several studies have evidenced the role of lysosomal degradation in focal adhesion recycling during migration. Autophagy-deficient cells have a reduced migration rate and increased focal adhesion number and size [215,216]. Moreover, the autophagic receptor NBR1 has been shown to promote focal adhesion disassembly and turnover through the autophagy pathway [215]. The essential role of autophagy in neuronal migration has been further confirmed in neuroblasts deficient for ATG5 or ATG12, which exhibit an accumulation of focal adhesion molecules such as Paxillin [217]. As a component of focal adhesion, the integrin steady-state is dictated by autophagy [218]. Indeed, VPS18 depletion leads to an upregulation of β1 integrin. Mice lacking VPS18 show neural migration defects in the cerebral cortex, hippocampus, and cerebellum [219]. As seen for ATG5 and ATG7 [93,94], VPS18 deficiency results in embryonic or early postnatal lethality and its neuronal-specific depletion leads to postnatal lethality and growth retardation [219], confirming the essential role of autophagic degradation during neuronal development.

During adult neurogenesis, autophagy has been found to be positively regulated by let-7 miRNA, permitting radial migration of newly formed olfactory bulb interneurons [220]. Let-7 downregulation prevents radial migration and maturation, which can be rescued by Beclin1 overexpression [220]. However, the RING finger E3 ligase RNF216 mediates Beclin1 ubiquitin-dependent degradation and promotes cell migration of immortalised gonadotropin-releasing hormone expressing neurons. RNF216 inhibition or autophagy activation inhibits neuronal migration [221]. It is nevertheless interesting to note that Beclin1 downregulation alone does not affect cell migration, suggesting another non-canonical role of Beclin1 during neurogenesis [221].

Several mutations of the autophagy pathway cause NDD in humans (Figure 3). MTOR mutations have been identified in patients affected by focal malformations of cortical development (FMCDs), a heterogeneous group of cortical abnormalities associated with epilepsy, intellectual disability, developmental delay, and autism [222,223,224,225,226,227]. MTOR mutations cause defective autophagy and disrupted migration of cortical neurons [228]. Patients carrying VPS15 mutations exhibit a wide range of symptoms, including cortical and optic nerve atrophy, cortical dysplasia, intellectual impairment, spasticity, ataxia, psychomotor delay, and late-onset epilepsy [31]. A VPS15 mutation has been shown to compromise the function of the PI3KC3 complex, leading to an accumulation of autophagic substrates [31]. RNF216 mutations have been identified in patients affected by Gordon Holmes syndrome, characterised by ataxia, dementia, and hypogonadotropic hypogonadism [229].

### 4.3. Neurite Outgrowth

The involvement of autophagy in neurite outgrowth has been evidenced for both axons and dendrites [230].

Autophagy can act as a negative regulator of axonal outgrowth. ATG12 is downregulated by the miRNA Mir505-3p, which inhibits autophagy, therefore promoting neural polarity and increasing axonal length and axonal branching [231]. Mir505-3p depletion increases the number of AVs and lowers the density of mitochondria, indicating increased mitophagy [231]. Axonal outgrowth, a high energy-demanding process, requires functional mitochondria throughout the axon, in order to provide a large amount of ATP [232,233]. Thus, it has been proposed that Mir505-3p locally downregulates axonal autophagy to maintain a sufficient mitochondria pool to promote axonal extension and branching [231]. This is consistent with previous studies reporting an excessive elongation of axons after autophagy inhibition through the depletion of ATG7 in cortical neurons [234] and of ATG2, ATG13, or ATG9 in *C. elegans* [137]. In addition, STX17 depletion has been shown to induce a reduction of axonal length in an AD mouse model [235].

Finally, several other studies reveal the opposing roles of autophagy on neurite outgrowth. Thus, ATG7 and/or ATG9 deficiency in mice does not increase axonal length but does impair nerve fibre formation with a larger contact site in axon terminals [236]. Moreover, pharmacological inhibition of autophagy decreases neurite length and branching complexity [237] of dorsal root ganglion neurons, and leads to a loss of neuronal polarity in hippocampal neurons, while its activation stimulates axonal growth [238]. During hypothalamus development, ATG7 depletion in neurons producing pro-opiomelanocortin induces alterations of neuronal projections that decrease their ability to extend axons during postnatal development [239].

The role of autophagy in dendrite outgrowth is more poorly studied than in axons. However, some studies have evidenced its crucial involvement in dendritic arborisation. AP-2, a key factor in ATG9 localisation [240] as for AP-4, has been described as an adaptor protein forming a complex between LC3 and dynein/dynactin, regulating autophagosome retrograde transport [241]. AP-2 depletion or downregulation leads to defective retrograde transport of AVs and impairs dendritic arborisation both *in vitro* and *in vivo* [241,242]. Interestingly, while AP-4 deficiency induces ATG9 mislocalisation in the axon, ATG9 in dendrites remains unaltered [135], confirming a compartment-dependent regulation of autophagy. The role of autophagy in dendrites is controversial. Downregulation of ATG5 and ATG7 was found to result in enlarged dendritic trees [243], while another study showed that basal autophagy is required for dendritic growth and branching [244]. Surprisingly, the latter study also showed that Atg1 (ULK1) overexpression decreases dendritic tree growth and terminal branching [244], indicating that both insufficient and excessive autophagy are deleterious for neuritic development.

Mutations and dysregulations of autophagy proteins may lead to neurodevelopmental and neurodegenerative diseases due to abnormalities in neurite outgrowth.

Neurodevelopmental abnormalities with neural tube closure defects, such as spina bifida, have been associated with the duplication of genomic segments containing Mir505-3p [245]. AP-4 mutations are causal for NDDs such as Cerebral Palsy and HSP [246,247], characterised by defects in axon outgrowth and neurodegeneration. Mimicking AP-4 loss-of-function in disease, AP-4 depletion was shown to reduce axonal ATG9A, leading to decreased autophagosomes in axons that may underly defective axonal extension [79,80,135]. Mutations of WIPI proteins cause NBIA, a NDD for which patients exhibit reduced white matter volume [248].

### 4.4. Synapse Development and Plasticity

**Figure 4:**
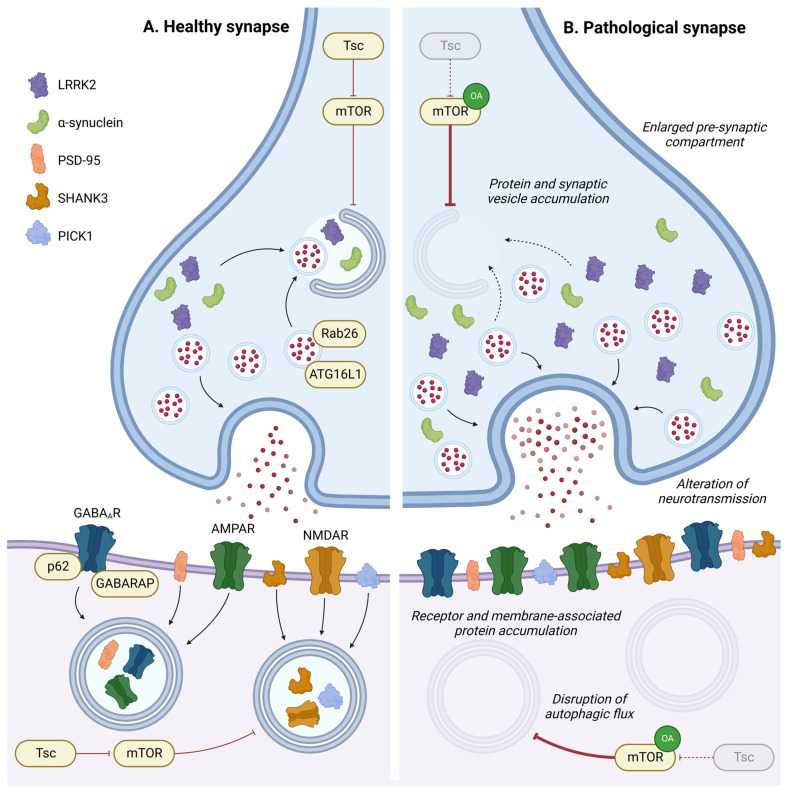
Role of autophagy in synapse homeostasis. In pre- or post-synaptic compartments, autophagy has a key role in regulating synaptic proteins or vesicles and is implicated in synaptic plasticity in response to environmental stimulation. Disruption in the autophagic process leads to accumulation of intracellular components, neurotransmitter receptors, and membrane-associated proteins in pre- or post-synaptic compartments, leading to an enlargement of the pre-synaptic compartment and alterations in neurotransmission. OA: Overactivation; GABA_A_R: gamma-aminobutyric acid receptor; AMPAR: α-amino-3-hydroxy-5-methyl-4-isoxazolepropionic acid receptor; and NMDAR: N-methyl-D-aspartate receptor.

Autophagy at synapses has a specific mode of regulation, as described in the previous section (Figure 2C). In this specific compartment, autophagy and neuronal activity are reciprocally regulated. In this section, we will specifically discuss the roles of autophagy in regulating synaptic plasticity, from synaptogenesis to the recycling of neurotransmitter receptors (Figure 4).

Synaptic defects and malformations have been reported *in vivo* as a result of genetic autophagy impairments. In *C. elegans*, autophagy is required for clustering synaptic vesicle proteins and promoting presynaptic assembly in interneurons during development [137]. In the *D. melanogaster* learning centre (i.e., mushroom bodies), autophagy inhibition increases the size of presynaptic active zones [249]. Conversely, mutations in different ATG genes in *D. melanogaster* induce a reduction of neuromuscular junction (NMJ) size, while induction of autophagy by Atg1 (ULK1) overexpression leads to NMJ overgrowth [250]. *Atg7^−/−^* flies present an increase in ELKS-family protein Bruchpilot (BRP), an essential building block of the active zone scaffold, and an increase in active zone size leading to memory impairment [251]. In mice, motor neuron-specific deletion of *ATG7* induces structural and functional defects within the NMJ [252]. By crossing ATG7-depleted mice with ALS mice, autophagy inhibition was shown to accelerate early neuromuscular denervation in pathological mice [252].

Altogether, these findings demonstrate that a finely regulated basal level of autophagy is required to structure synapses, both between neurons in the central nervous system and at the NMJ in the peripheral nervous system.

Autophagy deficiency in dopaminergic neurons by ATG7 depletion increases the size of dopaminergic synaptic terminals and induces striatal dopaminergic innervation, leading to increased dopaminergic neurotransmission [253]. This result indicates the involvement of autophagy in neurotransmission regulation at presynaptic sites. In agreement with this, stimulation of autophagy by rapamycin has been shown to decrease the number of synaptic vesicles through autophagic degradation in mice [253]. Moreover, ATG16L1 has been found to be recruited at the axonal terminal by the GTPase Rab26, which is enriched in synaptic vesicle clusters to mediate their autophagic degradation [254].

Several studies have pinpointed the roles of autophagy in the postsynaptic region. After neurogenesis, dendritic spines can be removed during postnatal development, in a process called spine pruning, which only stabilises specific synapses. Synapse morphology and dendritic spine remodelling rely on the autophagic degradation of key postsynaptic proteins such as SHANK3 (SH3 and multiple ankyrin repeat domains protein 3), PSD-95 (postsynaptic density protein 95), and PICK1 (protein interacting with C kinase 1) [255]. Autophagy activation in fragile X mice, which exhibit mTOR overactivation, allows for autophagy-dependent degradation of PSD-95 and corrects spine morphology and cognition deficits [256]. Overall, activation of autophagy signalling represents a strategy to normalise developmental dendritic spine pruning defects and social behaviours [257]. However, inhibition of autophagy via mTOR causes spine pruning defects. Thus, tuberous sclerosis protein (Tsc) haplo-insufficient mice studies, frequently used as an ASD model, result in a constitutive overactivation of mTOR and cause postnatal spine pruning alterations [257]. Additionally, Tsc1-deficient neurons exhibit alterations in excitatory functions characterised by an enhancement of postsynaptic glutamatergic functions due to an augmentation in the number of synapses [258]. Interestingly, Tsc1 deficiency in GABAergic interneurons of young adult mice results in a reduced number of synapses. This phenotype has been associated with a transient autophagy dysfunction in adolescent mice, presenting dysregulation in the expression of several autophagic markers (LC3-II, p62, ULK1) [259].

Autophagy also regulates neuronal activity via synaptic receptor recycling. The induction of autophagosome formation in the dendrites and dendritic spines of pyramidal neurons due to long-term depression contributes to AMPAR internalisation for lysosome degradation [195]. AP-4-deficient Purkinje cells and hippocampal neurons exhibit defective transport of AVs containing AMPAR, which accumulate in dystrophic axons [260]. Autophagy-mediated degradation of AMPAR in hippocampal neuron spines contributes to memory destabilisation [261], enabling a return to a labile state to reinforce or update original memories [262]. Autophagy may also play a key role in the formation of new memories. Indeed, impaired autophagy activity has been observed in old hippocampal neurons and is associated with memory decline, and the restoration of autophagy in these neurons is capable of reversing age-related memory deficits [263]. Moreover, different models of synaptic plasticity impairment have been used to investigate the neuroprotective effect of autophagy. Rapamycin treatment relieves the impairments of cognitive function in melanin-treated rats as well as anxiety symptoms in a hindlimb unloaded mice model for depression and anxiety [264,265]. Interestingly, the induction of autophagy rescues the synaptic defects by increasing NMDAR and PSD-95 expression [264].

The synaptic receptor for gamma-aminobutyric acid (GABA) is also dependent on the autophagic process. In *C. elegans*, the absence of presynaptic inputs induces GABA_A_ receptors but not acetylcholine receptor internalisation in autophagosomes for degradation [266]. Similarly, Beclin1-deficient mice exhibit an upregulation of GABAergic synaptic currents and long-term plasticity impairments in the neocortex and hippocampus [267]. It should be noted that in these mice glutamatergic signalling is downregulated, suggesting a direct or indirect regulation of glutamatergic receptor through autophagy. *Ulk2^+/−^* mice have an upregulated expression of p62, predominantly found in pyramidal neurons of the prefrontal cortex [268]. These neurons show an imbalanced excitatory–inhibitory neurotransmission, due in part to a selective downmodulation of GABA_A_ receptor surface expression in pyramidal neurons, leading to behavioural deficits related to schizophrenia (SZ) and bipolar disorders (BD) [268]. In autophagy-deficient neurons, p62 accumulation induces a disruption of GABA_A_ receptor trafficking to the cell surface, revealing the physiological role of autophagy in regulating GABA signalling [268,269]. Thus, reduced autophagy is a potential mechanism behind the reduced inhibitory inputs observed in neurodevelopmental and neuropsychiatric disorders such as SZ, BD, and ASD.

The role of autophagy in synapses is not limited to neurons but also extends to glial cells. Mice with a microglia-specific *ATG7* deletion present socio-behavioural defects and repetitive behaviours, which are characteristic features of ASDs. These mice also present an increase in dendritic spines and altered connectivity between brain regions, indicating defects in synaptic refinement [270]. Conversely, brain-derived neurotrophic factor (BDNF) signalling regulates autophagy, through transcriptionally inhibiting key components of the autophagic pathway. Genetic ablation of BDNF in the neural lineage results in an increase in autophagy in the adult brain and is also associated with severe synaptic defects [255].

In this work, we reviewed the roles of autophagy in sustaining both the structural integrity and functionality of the synapses, whose dysregulations underly the pathogenesis of neurodevelopmental and neurodegenerative diseases. Indeed, Tsc mutations lead to an autosomal dominant disorder characterised by seizures, mental retardation, and autism, due to neurodevelopment abnormalities [271]. Furthermore, gene expression analyses on post-mortem brain samples of SZ patients suggest possible deficits in autophagic signalling with a downregulation of ATG7 and ATG3, implicated in synaptic plasticity [272]. Reduced inhibitory inputs have been described in psychiatric conditions. Olfactory neurons from SZ and BD patient biopsies display increased p62 protein levels with LC3-II accumulation, revealing attenuated autophagy in patients [268]. The levels of insoluble GABARAPs are higher in at least a subpopulation of ASD patients [269], confirming the role of autophagy-mediated sequestration of GABA receptors in psychiatric disorders. Other genetic studies have previously identified copy number variations of ATG7 or GABARAPs in patients with ASD or ID [45,273]. Interestingly, mutations of the postsynaptic proteins SHANK3, PSD-95, and PICK1 are implicated in ASD, and, similarly, ASD patients also exhibit mutations in autophagy genes [45]. Moreover, AV accumulation, particularly in its immature forms, has been observed in dystrophic neurites and brain synapses among AD patients [172]. Failed autophagosome formation has been found in behavioural and psychological symptoms of dementia, present in 95% of AD.

## 5. Concluding Remarks and Perspectives

Autophagy is a cellular pathway that is essential for maintaining cellular homeostasis in neurons. This process is orchestrated by a complex set of events, involving various core autophagy-related (ATG) proteins and many regulators. In this review, we presented our current knowledge of the different steps underpinning the process of macroautophagy and the numerous neurodevelopmental/neurodegenerative diseases caused by mutations in autophagic components. We emphasised how the neuron has elaborated a fine-tuned and specialised system in different compartments to respond to and to sustain specific needs in the synapse, the axon, and the soma. The recent field of neuronal autophagy has mainly been investigated in the axon, and future challenges will involve the further examination of the dynamics and function of macroautophagy within the somatodendritic compartment and the need to further detail the coupling of AV dynamics and transport. This will be essential to unveil the spatial roles of autophagy in polarised cells and to shed light on the complexity of this degradative pathway in health and disease. Indeed, efforts in both fundamental and translational research will certainly expand our current knowledge of the key roles of autophagy, as presented in this review, affecting the various developmental processes and the maintenance of plasticity and neurotransmission in adults in health and disease. Considering the wide range of neurological disorders due to mutations in autophagy genes, deciphering the neuronal specificity of autophagy establishment, regulation, and function in a spatial resolution across the nervous system is of great biomedical importance, potentially paving the way to more specialised therapeutic approaches for patients.

## Abbreviations

AD	Alzheimer’s disease
ALFY	Autophagy-linked FYVE protein
ALS	Amyotrophic lateral sclerosis
AMBRA1	Activating molecule in BECN1-regulated autophagy protein 1
AMPA (R)	α-amino-3-hydroxy-5-methyl-4-isoxazolepropionic acid (receptor)
AP	Adaptor protein
ASD	Autism spectrum disorder
ATG	Autophagy-related proteins
AV	Autophagic vesicle
BPAN	β-propeller protein-associated neurodegeneration
BD	Bipolar disorders
CHMP	Charged multivesicular body protein
CMT	Charcot–Marie–Tooth disease
DD	Developmental delay
DQ-BSA	dye quenched-bovine serum albumin
EndoA	Endophilin A
EPG5	Ectopic P granules protein 5 homolog
Eva1a	Epithelial v-like antigen 1
FIP200	Focal adhesion kinase family interacting protein of 200 kDa
FTD	Fronto-temporal dementia
FYVE	Fab1, YOTB, Vac1 and EEA1 domain
GABA	Gamma-aminobutyric acid
GABARAP	Gamma-aminobutyric acid receptor-associated protein
GAN	Giant axonal neuropathy
HD	Huntington’s Disease
HOPS	Homotypic fusion and protein sorting
HAP1	Huntingtin-associated protein 1
HSP	Hereditary Spastic Paraplegia
HSPB1	Heat shock protein beta-1
HTT	Huntingtin
ID	Intellectual disability
JIP	JNK-interacting protein
LAMP	Lysosomal membrane protein
LC3	Microtubule-associated protein light chain 3
LRRK2	Leucine-rich repeat serine/threonine-protein kinase 2
MT	Microtubules
mTOR	mammalian target of rapamycin
NBIA	Neurodegeneration with brain iron accumulation
NBR1	Next to BRCA1 gene 1 protein
NDD	Neurodevelopmental disorders
NDP52	Calcium-binding and coiled-coil domain-containing protein 2 (Calcoco2)
NMDA (R)	N-methyl-D-aspartate (receptor)
NSCs	Neural stem cells
NPCs	Neural progenitor cells
OPTN	Optineurin
PD	Parkinson’s disease
PI3KC3	class III phosphatidylinositol 3-kinase
PI3P	phosphatidylinositol-3-phosphate
PICK1	Protein interacting with C kinase 1
PSD-95	Postsynaptic density protein 95
RNF	Ring Finger Protein
SCA	Spinocerebellar ataxia
SHANK3	SH3 and multiple ankyrin repeat domains protein 3
SNAP	Synaptosomal-Associated Protein
SNARE	SNAp Receptor
SZ	Schizophrenia
Tsc (1)	Tuberous sclerosis protein (1)
ULK	Unc-51-like kinase
VAMP	Vesicle-associated membrane protein
VPS	Vacuolar protein sorting
WIPI	WD repeat domain phosphoinositide-interacting protein
